# Examining key design decisions involved in developing a serious game for child sexual abuse prevention

**DOI:** 10.3389/fpsyg.2014.00073

**Published:** 2014-02-04

**Authors:** Colleen Stieler-Hunt, Christian M. Jones, Ben Rolfe, Kay Pozzebon

**Affiliations:** Faculty of Arts and Business, Engage Research Cluster, School of Social Sciences, University of the Sunshine CoastSippy Downs, QLD, Australia

**Keywords:** video games, serious games, serious game design, child protection, sexual abuse, personal safety program

## Abstract

This paper presents a case study of the key decisions made in the design of *Orbit*, a child sexual abuse prevention computer game targeted at school students between 8 and 10 years of age. Key decisions include providing supported delivery for the target age group, featuring adults in the program, not over-sanitizing game content, having a focus on building healthy self-concept of players, making the game engaging and relatable for all players and evaluating the program. This case study has implications for the design of Serious Games more generally, including that research should underpin game design decisions, game designers should consider ways of bridging the game to real life, the learning that arises from the game should go beyond rote-learning, designers should consider how the player can make the game-world their own and comprehensive evaluations of Serious Games should be undertaken.

## Introduction

This paper presents key decisions in the development of a Serious Game for child sexual abuse prevention. Serious Games is an umbrella term used to encompass digital games designed for a purpose beyond entertainment. Serious Game designers aim to use qualities of digital game-play such as immersion, interaction, and engagement, to change people's thoughts or behaviors or help them learn something. Within the Serious Games field, there are a large number of overlapping game sub-groupings that have emerged organically over time (Sawyer and Smith, 2008). Those most relevant to this paper are Games for Health (“Games for Health,” 2013), Games for (social) Change (“Games for Change,” 2005) and learning games. Aside to the Serious Games movement, there is also a movement around games-based learning which involves using games, either entertainment games or Serious Games, to aid the learning process in formal learning environments such as schools and universities.

Proponents of using digital games as part of the learning process believe that games provide opportunities to “learn through doing” and in doing so promote understanding, motivation, and enjoyment (Kirriemuir and McFarlane, 2004; Gee, 2008; Klopfer et al., 2009; Shute et al., 2010). They also provide significant opportunities for social interaction between student players and between the student players and the teacher (Gee, 2003, 2008). In addition, games also provide immediate feedback, allow players to achieve at their own level in their own way and give players a sense of agency over the game environment (Salen and Zimmerman, 2004; Gee, 2008; Shute et al., 2010). However, teachers report difficulty in finding games of a high enough quality that meet both curriculum needs and players' expectations (Kirriemuir and McFarlane, 2004; Egenfeldt-Nielsen, 2005). Therefore, there is a need to produce high quality games that address curricula in meaningful ways.

The curriculum in this case study is child sexual abuse. Child sexual abuse is a significant societal problem. Whilst its prevalence is not known because a significant amount of abuse goes unreported, it is estimated that from 7 to 62 percent of women and from 3 to 19 percent of men have experienced some form of child sexual abuse on at least one occasion (Sanderson, 2004). People experiencing sexual abuse as children can experience negative impacts for the entirety of their lives (Lamont, 2011; Queensland Government, 2011). Individuals who have been sexually abused as children have a higher risk of psychological problems, suicide, drug and alcohol misuse, engaging in high-risk sexual behavior, homelessness, eating disorders and obesity, physical health problems and displaying aggressive, violent and criminal behavior (Mullen and Fleming, 1998; Access Economics, 2008; Lamont, 2011; Queensland Government, 2011). Access Economics estimates the annual cost for child abuse and neglect costs Australian society four billion dollars each year (Access Economics, 2008). Recovery from child sexual abuse is best facilitated by a supportive network of significant others (Mullen and Fleming, 1998). Children who are able to share information with a trusted adult, and who are believed, experience less impact than children who do not disclose the abuse. Therefore, the need exists to produce programs that effectively educate children and adults about sexual abuse and its prevention.

Evaluations of child sexual abuse prevention programs and serious games are limited. Only 2.6 percent of Australian child sexual abuse prevention programs are comprehensively evaluated (Sanderson, 2004), and until recently few serious games are rigorously evaluated (for example, Beale et al., 2006, 2007; Nudell et al., 2007; Kato et al., 2008; Kognito Interactive, 2009; Froschauer et al., 2010; Knight et al., 2010; Shute et al., 2010; Wrzesien and Alcañiz Raya, 2010; Muratet et al., 2011; Alamri et al., 2014). However, evaluating Serious Games is an emerging research area with a number of researchers working toward evaluation frameworks for evaluating aspects of Serious Games (for example, Ekanayake et al., 2010; Nacke et al., 2010) or Serious Games as a whole (for example, de Freitas and Oliver, 2006; De Freitas et al., 2010; Mayer et al., 2013). This paper intends to support rigorous evaluation through presenting key decisions in the evaluation of the child sexual abuse prevention serious game.

Part of our rationale for using a computer game to help children learn about child sexual abuse prevention is that computer games are the medium of this generation and most Australian children in our target age group play games (Brand et al., 2008).

Another reason for creating a game around this subject matter is that although teachers recognize the importance of child sexual abuse prevention, generally they do not feel confident with the subject matter (Scholes et al., 2012). It is intended that teachers can use this game as a basis for addressing this sensitive issue.

Furthermore, good games will engage the player and provide them with emotional experiences in a low risk environment through the narrative presented in the game and interaction with the game-world, game characters, and immersion in the audio-visual environment.

This paper presents a case study of the key decisions made in the design of *Orbit*, a child sexual abuse prevention computer game targeted at school students between 8 and 10 years of age. In Section Materials and Methods, we give an overview of the materials and methods used in this study. Section Results identifies the evidence underpinning the key design decisions. Finally, we discuss the implications this case study has for the design of Serious Games more generally.

## Materials and methods

Multidisciplinary research teams were established to compile reports and develop recommendations for (1) key messages for an effective sexual abuse prevention program, (2) effectively training teachers in sexual abuse prevention, (3) program delivery, support and data around incidence of reporting disclosure, (4) computer games and their use in education and (5) learning styles of boys and girls in our target age group. These reports were critiqued by an external reviewer who is an expert in child sexual abuse prevention as well as psychologists, counselors, and social workers.

These working group reports were used to develop a “theory of change” as suggested by Swain (2007). A “theory of change” articulates the holistic process of how the program aims to bring about change in its participants. This “theory of change” was further elaborated into a series of 54 individual learning objectives for children participating in the program and 80 individual learning objectives for adults involved in the program.

Subject matter experts in a range of fields including counselor practitioners working with children who have been sexually abused, Queensland Police Service and psychologists regularly consulted on the game's design. Focus groups and play-test sessions were also held with school children in our target age group throughout the design process.

In the early phases of designing the game, we used many different ideation techniques, including trying to find the “play space” in the topic as outlined by Klopfer et al. (2009), using the game-like stimulus-based ideation techniques developed by Paavilainen et al. (2009), thinking about our end users in terms of their play personalities (Brown, 2009), beginning each ideation session with a problem statement (Schell, 2008) and thinking of our game space as a system (Brathwaite and Schreiber, 2009; Klopfer et al., 2009). Taking more than a year of weekly meetings to come up with the ideas and develop them, we endeavored to use all elements of the game as meaningfully as possible to assist with the learning process.

The game that was developed is titled *Orbit. Orbit* is an adventure game with an interactive story line and a series of integrated mini-games. The story centers on Sammy, a spaceship who has become emotionally distant from its concerned crew. The crew consists of six aliens: Epsilon the navigator, Delta the scientist, Zeta the janitor, Tau the robotics' expert, Rho the security officer, and Chi the chef. The game begins with an earth child (the player character) beaming aboard the spaceship. The player is tasked with doing all he/she can to help Sammy. Throughout the game, the player character teleports trusted adults aboard from his/her life to help. Together, they unravel what has happened to Sammy. Gradually, Sammy learns what to do to start the recovery process. The game has five chapters entitled: (1) togetherness, (2) listening, (3) understanding, (4) belief, and (5) courage. Integrated within these chapters are four mini-games: *Robot Factory*, *“Need to Tell” machine*, *Speak Up*, and *Surveillance Footage*.

The majority of the game-play was designed by three game designers/researchers. The list of key design decisions was compiled by one of the game's designers and after interviewing the other two game designers.

## Results

Any creative project is the result of many decisions made during the design and development process. This results section describes some of the key game design decisions and justifies them with research evidence. These key decisions have been grouped into seven sections: Key decision 1: delivery of program to be supported by classroom activities, Key decision 2: longer duration program, Key decision 3: feature adults in the program, Key decision 4: not over-sanitizing content, Key decision 5: focusing on building healthy self-concept, Key decision 6: making the game engaging and relatable for all players, and Key decision 7: evaluate the program. These results are summarized in Table 1.

**Table 1:** **Key design decisions and their justification**.

**Key design decisions**	**Justifications**
**Key decision 1**. Delivery of program to be supported by classroom activities.	More effective child sexual abuse prevention programs are integrated into the school curriculum (Sanderson, 2004). Classroom activities provide more opportunities for discussion and reflection (Rispens et al., 1997).
**Key decision 2**. Longer duration program—design the *Orbit* program so that it takes place over several weeks.	Longer child sexual abuse prevention programs are more effective than one-off presentations (Hazzard et al., 1991; Finkelhor et al., 1995; Rispens et al., 1997; Sanderson, 2004).
**Key decision 3**. Feature adults in the *Orbit* program by providing: support and educative information for families, teachers, and community members. opportunities for adults to get involved in the program through special trusted adult logins and opportunities for side-by-side game-play with trusted adults. virtual representations of the players' trusted adults within the game through the use of avatar generators. The game requires the player to create virtual representations of five trusted adults from at least three different parts of their life. opportunities for players to reflect on the suitability of the adults in their support network through the game's narrative, game dialog boxes, and an in-game utility to facilitate further reflection.	Many child sexual abuse prevention programs put too much responsibility on children to protect themselves (Sanderson, 2004; Wurtele, 2009; Scholes et al., 2012). Most effective programs include parental involvement (Briggs and Hawkins, 1994; Wurtele, 2002; Sanderson, 2004) and community integration (Berrick and Barth, 1992; Wurtele, 2002). Although a significant societal problem, child sexual abuse is not well understood by many adults (Putnam, 2003; Tucci et al., 2006) and some common practices and stereotypes can assist perpetrators of child sexual abuse (Somer and Szwarcberg, 2001; Johnson, 2004; Sanderson, 2004). Identifying five adults a child can reliably turn to when requiring assistance is a recognized protective behavior for children (Wurtele, 2002; Queensland Government, 2012; Scholes et al., 2012). Subject matter experts consulting on the project felt it was important that children themselves were able to select and change their five trusted adults in their support network, they also felt that these adults should be from a variety of areas in the child's life and that the child should be encouraged to reflect on the suitability of adults in their trusted adult support network.
**Key decision 4**. Not over-sanitizing content by: using a rules-based understanding of sexual abuse. helping players understand perpetrator tactics helping players understand the barriers to telling. featuring the perpetrator as a main character in the game.	In an effort to avoid controversy and fear and anxiety in children, some child sexual abuse prevention programs over-sanitize content, so much so that they are ineffective (Finkelhor and Strapko, 1992; Sanderson, 2004; Tucci et al., 2006). Recognizing sexual abuse is a key part of many prevention programs (Wurtele, 2002). Some programs suggest children use feelings to ascertain whether a situation is okay (Wurtele, 2002) but this approach fails to recognize grooming/normalizing behaviors that may accompany sexual abuse and that sexual touching may feel good (Sanderson, 2004). Developing a rules-based understanding of sexual abuse helps to combat these issues. The scenarios used within the mini-games were based on reports of child sexual abuse collated by police and counselors. These scenarios served to help children understand the tactics that perpetrators use to abuse children and acknowledge the emotional and psychological barriers that often prevent children from telling adults about abuse (Somer and Szwarcberg, 2001; Putnam, 2003). Perpetrators of child sexual abuse are likely to use careful grooming strategies that instill trust and use their authority to abuse a child rather than perpetrate a sudden attack on a child (Smallbone and Wortley, 2001; Wurtele, 2002; Sanderson, 2004; Scholes et al., 2012) therefore the person who plays the abuser in the game's main storyline is liked and valued by the other characters in the game.
**Key decision 5**. Focus on building healthy self-concept.	Children who have a healthy self-concept are more likely to retain information presented in child sexual abuse prevention programs and are more likely to resist a perpetrator (Sanderson, 2004). Perpetrators report that they select victims who are “passive, troubled, lonely” children and use these characteristics to prevent them from telling an adult about the abuse (Budin and Johnson, 1989).
**Key decision 6**. Engaging and relatable for all players.	Many child sexual abuse prevention programs have been criticized for not catering well for boys (Asdigian and Finkelhor, 1995; Finkelhor and Dziuba-Leatherman, 1995; Sanderson, 2004; Scholes et al., 2012) whilst many mainstream games are criticized for alienating women and girls (Dietz, 1998). Also important is that the program consider children with disabilities (Briggs and McVeity, 2003; Sanderson, 2004) and children that have experienced or are experiencing abuse (Currier and Wurtele, 1996; Scholes et al., 2012). The game was set in a fantastical game environment with the ability to customize player game characters, earn rewards and personalize parts of the game environment (Malone, 1980; Hedden, 1998; Poremba, 2003; Dondlinger, 2007; Gibson et al., 2007).
**Key decision 7**. Evaluate the program.	Many child sexual abuse prevention programs are criticized for not being evaluated rigorously (Melton and Flood, 1994; Sanderson, 2004). This program will be evaluated using pre- and post- tests and a control group (Sanderson, 2004) using variations to What-If Situation Test (Wurtele et al., 1998) and the Children's Knowledge of Abuse Questionnaire (Tutty, 1995).

### Key decision 1: delivery of program to be supported by classroom activities

Sanderson's (2004) comprehensive research into the effectiveness of child-focused sexual abuse prevention programs found programs integrated into the school curriculum tended to be more effective. Furthermore, Rispens et al. (1997) suggests classroom activities allow for more discussion and reflection on program content. Other research findings indicate that older children retain child sexual abuse concepts better than children in preschool or early primary school (Finkelhor et al., 1995; Sanderson, 2004). Therefore, the *Orbit* program was developed to be integrated into the year 4 (children between 8 and 10 years of age) curriculum of Australian schools. The program consists of a computer game designed to be played individually by each child in the class and a set of lesson plans to be used by the teacher to clarify and extend game concepts.

### Key decision 2: longer-duration program

Child-focussed sexual abuse prevention programs range from one-off presentations to longer-duration programs (Sanderson, 2004), with longer programs proven to be more effective (Hazzard et al., 1991; Finkelhor et al., 1995; Rispens et al., 1997; Sanderson, 2004). Therefore, the *Orbit* program was developed so that the player could progressively develop key learnings and skills. The game provides about 6 h of game-play and is designed to be integrated into classroom activities over a period of 5–12 weeks. The game saves player progress as the player plays through the interactive story.

### Key decision 3: feature adults in the program

A criticism of many child sexual abuse prevention programs is that they put too much onus on children to be responsible for their own safety; when it is the responsibility of adults to protect children (Sanderson, 2004; Wurtele, 2009; Scholes et al., 2012). Therefore, researchers recommend that the most effective programs will include parental involvement (Briggs and Hawkins, 1994; Wurtele, 2002; Sanderson, 2004) and community integration (Berrick and Barth, 1992; Wurtele, 2002). The *Orbit* program includes adults by (a) providing support and educative information for families and community members, (b) having ways that adults can get involved in the program, including playing the game alongside the child, (c) including virtual representations of adults in the players' game-play and (d) encouraging students to reflect on the suitability of the adults in their support network.

#### (a) Providing support and educative information for families, teachers, and community members

Recognition of child sexual abuse as a significant societal problem is relatively recent, with its prevalence only beginning to be realized as recently as the late 1970s (Putnam, 2003). Therefore, child sexual abuse is not well understood by many adults (Tucci et al., 2006) and some common societal practices such as children being expected to obey adult authority (Somer and Szwarcberg, 2001), stereotypes of perpetrators as scary strangers that attack a child suddenly (Sanderson, 2004) and the culture of secrecy around child sexual abuse (Johnson, 2004) can assist perpetrators of child sexual abuse. In addition, many adults do not know how to respond to a disclosure of child sexual abuse (Tucci et al., 2006) and teachers report feeling concerned about teaching child sexual abuse prevention programs (Scholes et al., 2012). Therefore, the *Orbit* website (www.orbit.org.au) includes information for adults on how to respond to disclosures of sexual abuse, general information about child sexual abuse, information about the game and key learnings and ideas for discussing these further with children. The website also contains specific information for teachers about how to use the game in the classroom and provides lesson plans that can be used.

#### (b) Providing opportunities for adults to get involved in the program

*Orbit* allows trusted adults to be directly involved in the program. An adult login code is generated each time the player character generates a new trusted adult. The player gives this code to their trusted adults along with an information slip. The slips explain what the game is, how to access the trusted adult section of the website and how to respond if a child discloses abuse to them. The trusted adult can use the code to log in to the *Orbit* website to find out more about what the player is learning in the game and leave positive messages for the player. The game further encourages positive interaction between the player character and their trusted adults by providing two mini-games that trusted adults can play side-by-side (two people at one computer) with the child player.

#### (c) Providing virtual representations of the players' trusted adults within the game

Proactively identifying five adults whom a child can reliably turn to when they need assistance is a recognized protective behavior for children (Wurtele, 2002; Queensland Government, 2012; Scholes et al., 2012). In addition, our subject matter experts recommended that these five adults come from a variety of areas of the child's life (e.g., family, school, sport, club) so that the child will be more likely to feel they have an adult they can turn to, no matter where they are and that the child is not selecting adults only from the environment where abuse may be occurring. Therefore, the *Orbit* game scaffolds the construction of the player's personal support network of five trusted adults. During each of the game's five chapters, a crew member asks the player character to teleport aboard the spaceship an adult from their life. In chapters one and two the player character is asked to teleport aboard an adult from their family and school, respectively. In chapter three the player character is asked to teleport aboard an adult from somewhere other than their family and school (e.g., sporting team, church, children's club) and in Chapters 4 and 5, the player character is asked to teleport aboard an adult from any of the aforementioned categories. The player uses an avatar generator to create a visual representation of each trusted adult character.

#### (d) Providing opportunities for players to reflect on the suitability of the adults in their support network

Our subject matter experts recommended that children reflect on the qualities of the relationship they have with their trusted adults and know that the choice of these trusted adults is in their control and that they can change their trusted adults at any time. To this end, we (1) constructed the game's narrative around trusted adults, (2) added “security warnings” to the in-game teleportation devices to say “Only beam aboard trusted adults who do not break the body rules” and (3) developed an in-game utility that the player could use to personally reflect on the qualities of the relationship they have with each of their trusted adults.

The game's narrative was themed around the key qualities that trusted adults should have. Each game chapter was named after one of these key qualities— “Chapter 1: Togetherness,” “Chapter 2: Listening,” “Chapter 3: Understanding,” “Chapter 4: Belief,” and “Chapter 5: Courage.” Once the trusted adult character is teleported aboard the spaceship, each trusted adult character helps the player character complete a chapter of the game. As the player constructs their support network by one trusted adult per chapter, Sammy, the child spaceship also rebuilds its support network of trusted adults, one crew member at a time. In chapter 1 Sammy learns that Delta, the spaceship's scientist, will always be there for Sammy, in chapter 2 Sammy learns that Zeta, the spaceship's janitor, will always listen to Sammy, in chapter 3 Sammy learns that Tau, the spaceship's robotics' expert, will always understand Sammy, in chapter 4 Sammy learns that Rho, the spaceship's security officer, will always believe Sammy, and in chapter 5 Sammy learns that Chi, the ship's chef, will always stand up for Sammy. These qualities are further reinforced in the *Speak Up* mini-game, in which the trusted adult player character has five special abilities, togetherness, listening, understanding, believing, and courage, that help the players navigate the platforms and solve the puzzle for each level.

### Key decision 4: do not over-sanitize content

A criticism of some sexual abuse prevention programs is that they are ineffective because they sanitize the content of the program in order to avoid controversy (Sanderson, 2004). This is understandable, since learning about child sexual abuse can induce fear and anxiety in children (Finkelhor and Strapko, 1992) and child sexual abuse can be a confronting topic even for adults (Tucci et al., 2006). However, there is no point having an ineffective program and therefore we endeavored to make the *Orbit* program positive, practical, and effective. Therefore, the program addresses potentially sensitive concepts such as “what is child sexual abuse,” “the tactics used by perpetrators of sexual abuse” and “barriers to telling about sexual abuse.”

#### (a) Rules-based understanding of sexual abuse

Recognizing sexual abuse is a key part of many sexual abuse prevention programs (Wurtele, 2002). However, child sexual abuse can take many forms and in some cases the difference between what is and isn't sexual abuse can be difficult even for adults to comprehend, so helping children recognize sexual abuse is potentially problematic due to children's level of cognitive development, societal norms around what is appropriate for children to learn about sex and the moral imperative of wanting to protect the innocence of children and not wanting them to be unnecessarily fearful.

Some child sexual abuse prevention programs tell children to use their feelings about a situation to tell them whether what is happening is okay (Wurtele, 2002). However, this kind of approach fails to recognize grooming behaviors that may accompany sexual abuse and that sometimes sexual touching may make the child feel good (Sanderson, 2004). To this end, we adopted a rules-based approach to understanding what sexual abuse is and how to respond to it. The player is introduced to the terms “private parts” and “breaking the body rules” in the first chapter of the game. In subsequent chapters it promotes “telling” trusted adults as the best course of action if someone breaks the body rules.

The *Robot Factory* mini-game first introduces this rules-based understanding of child sexual abuse. In this mini-game the player assembles robots by dragging the non-private body parts (head, arms, legs, and stomach) onto a blueprint of a robot but lets the private parts of the robot (mouth, chest, and area covered by the underpants) roll off a conveyor belt into a private parts section. Once the public parts of the robot have been assembled, the player places the partially-assembled robot in an assembly queue. The robot goes into a private dressing room, where it affixes its own private parts and the robot puts its “clothes” (paint) on. Therefore, the core activity of the mini-game requires the player to distinguish between the parts of the body that are private and those parts of the body that are not private.

#### (b) Understanding of perpetrator tactics

Children should to be aware of the tactics that perpetrators use to abuse, and they should understand that abuse is never the fault of the child. In addition children need to be able to identify the types of situations that should be communicated to their trusted adults. Some perpetrator tactics were enacted in the main storyline of the game. However, the main storyline only deals with one case of sexual abuse, albeit in detail, so the *“Need to Tell” Machine* and the *Speak Up* mini-games are introduced in chapters three and four, respectively, to help children understand the variety of tactics that perpetrators use to abuse children. The 55 abuse scenarios in these mini-games were based on reports of child sexual abuse collated by the Queensland Police Service and the collective experiences of counselors from Sunshine Cooloola Services Against Sexual Violence Inc. who work with children who have been sexually abused.

The *“Need to Tell” Machine* is introduced to the player as a communication system Sammy once used to tell the spaceship crew about “need to tell” situations. Throughout this mini-game a member of Sammy's crew, Rho, acts as a pedagogical agent guiding the activity of the player (Moreno and Mayer, 2007). Rho asks the player character to retrain the machine so that it will work properly again. In each level of the retraining, there are two phases of game-play followed by a debrief. Using a quiz-based mechanic, the player is given a number of scenarios and is instructed to tag each scenario as either “need to tell” or “do not need to tell.” The scenarios are written as text messages, audio recordings, voicemail messages or surveillance footage and communicated to the player using both on-screen text and voice to promote multimodal learning (Moreno and Mayer, 1999). In phase 2 of the game, the child must tell their trust adult those scenarios which they have tagged as “need to tell.” This phase of the game uses a “Guitar Hero” style music and rhythm mechanic. Each level focuses on a different offender tactic: bribes, tricks, secrets, coercion, making the child think no one will believe them, grooming, making the child think they wanted it, isolation, making the child think they are special and threats.

#### (c) Barriers to telling

Although it is widely acknowledged that emotional and psychological barriers prevent children from telling adults about abuse (Somer and Szwarcberg, 2001; Putnam, 2003), information about these barriers is not typically included in child sexual abuse prevention programs. These barriers include overcoming the influence of the abuser(s) and their power, pressure and control; and fears about what will happen if they tell (e.g., Will the person go to jail? Will I lose my family?). Our subject matter experts felt that these should be included as part of the program. This information was dealt with in the main storyline of the game and in the *Speak Up* mini-game.

Each level of the *Speak Up* mini-game explores a scenario where an alien child has been sexually abused. The player characters (child player character and trusted adult player character) are tasked with breaking down an “invisible” wall (barriers to telling), brick-by-brick. They do this by working together. The child player character picks up objects belonging to the alien child. These objects explain what the alien child is thinking or feeling and explain why they are not telling their trusted adults about their “need to tell” situation. Meanwhile, the trusted adult player character picks up stars. The stars provided advice on how to deal with the thoughts and feelings that the alien child is having.

#### (d) Representing the perpetrator

We purposefully depicted the person who sexually abused Sammy as an important part of the crew and as someone who seemed like a genuinely nice person. We avoided the stereotypical “baddy” representations often used in games because research shows that most adult perpetrators of sexual abuse will seem trustworthy to the child and their family. Furthermore, stereotypes of “evil-looking” people are unhelpful to understanding the nature of child sexual abuse because perpetrators are more likely to use careful grooming strategies that instill trust and use their authority to abuse a child rather than perpetrate a sudden attack on a child (Smallbone and Wortley, 2001; Wurtele, 2002; Sanderson, 2004; Scholes et al., 2012). We also tried to make the perpetrator look as non-descript as possible by attempting to make the character be of indeterminate age and have no distinguishing physical features. Early in development, we shared concept art of our characters with focus groups of children from our target age group and asked them to pick which character would be the “baddy.” Across six focus groups, we found that only one child suggested that the perpetrator character might have been part of a group of “baddies” in the game.

In addition, the 55 abuse scenarios used across mini-games characterized a variety of perpetrators (e.g., gender, age) and victims (e.g., gender, age), in a number of different power relationships (e.g., authority figure, family connection, stranger).

### Key decision 5: focus on building healthy self-concept

Children who have a healthy self-concept are more likely to retain information presented in child sexual abuse prevention programs and are more likely to resist a perpetrator (Sanderson, 2004). In addition, perpetrators report that they select victims who are “passive, troubled, lonely” children and use these characteristics to prevent them from telling an adult about the abuse (Budin and Johnson, 1989). Therefore, the *Orbit* game has a number of features designed to help build and reinforce a healthy self-concept including problem solving elements, “I am good at” boards, in-game rewards which can be used to decorate their bedroom and the living room on-board the spaceship, and the game's story models that children are important with strong statements such as, “nothing is more important than your safety,” appearing regularly in the script.

### Key decision 6: engaging and relatable for all players

Child sexual abuse prevention programs need to be engaging and relatable to all segments of the target audience (Scholes et al., 2012). We especially wanted to ensure that the game catered for both girls and boys—many child sexual abuse programs have been criticized for not catering well for boys (Asdigian and Finkelhor, 1995; Finkelhor and Dziuba-Leatherman, 1995; Sanderson, 2004; Scholes et al., 2012) and many mainstream games are criticized for alienating women and girls by presenting inappropriate portrayals of women (Dietz, 1998). We also considered how the program may impact children who have been or are currently being sexually abused (Currier and Wurtele, 1996; Scholes et al., 2012) and children with disabilities, who are at greater risk of being abused because they rely on adults more for their care (Briggs and McVeity, 2003; Sanderson, 2004). We did this by using a genre of game that was engaging to both boys and girls, setting the game in a fantastical environment, providing character and game-world customizations and making mini-game sexual abuse scenarios representative of our target audience.

We decided to use a fantastical setting for the game rather than a real-life setting as a fantastical game environment would be both appealing to players (Malone, 1980; Hedden, 1998; Gibson et al., 2007) and could also be used to effectively introduce players to these sensitive sexual abuse prevention concepts without feeling threatened by them. In addition, the distance from reality that a fantastical environment provides makes the game more appropriate for children who have been sexually abused in the past or are currently being sexually abused.

The ability to customize game characters and game environments is one way to give players agency, a sense that their decisions in the world matter (Poremba, 2003). The *Orbit* game offers players the ability to customize their own avatar and each of their trusted adults avatars so that they can create characters they can identify with. Character customizations included a wheelchair avatar. The player can also personalize the living space and their bedroom on the spaceship by changing color schemes and adding furniture and other objects to the room. Such objects are regularly given to the player for achieving game milestones. These are part of the rewards and goal system that serves to motivate players (Dondlinger, 2007). Another significant game customization is that, the gender of Sammy, the child spaceship character that the game's narrative centers around, is always the same gender as the player character, thus helping the player to identify a little more with Sammy and what Sammy is going through.

Across the levels of the two mini-games featuring sexual abuse scenarios, we made an effort to present both boys and girls as victims of abuse and vary the perpetrators, nature of the abusive situations and the trusted adults. Generally the perpetrator of abuse was male as this fits with statistics about the nature of child sexual abuse (Finkelhor, 1999), however, there were two scenarios where a woman was the perpetrator of abuse. We also included a scenario where the sexual abuse victim had a disability.

### Key decision 7: evaluate the program

Many child sexual abuse prevention programs are criticized for not being evaluated rigorously (Melton and Flood, 1994; Sanderson, 2004). We have designed a rigorous evaluation which will commence in late 2013 and involves pre- and post- tests and a control group (Sanderson, 2004). We will test whether this learning transfer has occurred by using slight variations to the What-If Situation Test (Wurtele et al., 1998) and the Children's Knowledge of Abuse Questionnaire (Tutty, 1995). The What-If Situation Test presents children with a number of situations and asks them to explain how the child should respond. The Children's Knowledge of Abuse Questionnaire evaluates children's learning of key concepts taught in most sexual abuse prevention programs (Tutty, 1995).

## Discussion and recommendations

In this section we reflect on the information provided in the Results section of this paper and combine it with other games literature to establish a list of five key recommendations for those wishing to create learning games.

### Research underpinning game design decisions

Our first recommendation is that research underpins the entire game design process. This needs to start well before design work begins on the game and continue right through to the game's evaluation. The research should include formal sources such as evidence-based research literature and informal sources such as subject matter experts (Swain, 2007).

In the case of *Orbit*, prior to designing the game, multi-disciplinary teams conducted a thorough review of the relevant research literature and a consultative team of subject matter experts was established prior to designing the game. This early research underpinned the game's learning objectives that were collaboratively developed with subject matter experts. Subject matter experts also regularly provided input into the game's design (Zyda, 2005; Swain, 2007).

Involving subject matter experts in the design of a game is challenging for both the game designers and subject matter experts (Kelly et al., 2007), as game designers do not necessarily have a full appreciation of the subject matter and subject matter experts do not have a full appreciation of the potential of using games for learning.

### Consider ways to bridge game to real life

Games do not exist in a vacuum, they are a part of culture more broadly (Salen and Zimmerman, 2004). Whilst with many games, the game permeates culture organically when game fans create and participate in discussion groups, fan sites, fan fiction, and other creative works such as Machinima (films created from game footage) (Berkeley, 2006), increasingly, entertainment game makers are purposefully providing tools to create original works like this to build community around a game (Thomas, 2004).

Many health games want to help people achieve long term behavioral change. Support from others can be a useful way of achieving these behavioral goals. Therefore, games with behavioral change objectives should consider how they can involve significant people from the player's life in the game. Our game used a combination of modeling behaviors, bringing the people into the game as a game character using an avatar generator, providing opportunities for side-by-side play with trusted adults and providing discussion prompts for parents via the game website and the trusted adult cards. Getting other people to play the game and possibly interact through the game also makes the game a shared experience that fellow players can discuss with each other either face-to-face or via social media or using other communication mediums.

There are other ways to bridge game play to the player's real life. Increasingly, developers of entertainment games are considering how teachers can use their games in the classroom. For example, in 2013 the makers of *Sim City*, announced that they were providing a website for teachers to share lesson plans based on the game (Hagen, 2013). Providing accompanying lesson plans for games fit well with games-based learning philosophies as the classroom provides opportunities to discuss, reflect on and debrief issues raised in the game (Crookall, 2010; Kriz, 2010).

Other examples of how digital games can bridge game-play to real life include emailing a politician about an issue addressed in the game, providing a facility to donate to a cause (Swain, 2007) and requiring players to take action in their real life to participate in the game (for example, Eklund et al., 2007; Solomon et al., 2012).

### Where possible, go beyond rote-learning

In the past many educational games have been developed using rote-learning. Usually games like this will use a quiz - based game mechanic. Some game scholars criticize games of this style because they fail to live up to learner expectations of games (Kirriemuir and McFarlane, 2004; Egenfeldt-Nielsen, 2005). These styles of games tend to be used when they are looking to improve response times to problems (e.g., number facts) or to check for the learning of facts.

Despite many games' scholars calling for educational games to go beyond rote learning, this can be challenging, as it is easy to “prove” traditional ideas of learning by asking someone an exam-style question and having them respond correctly. Klopfer et al. (2009) suggest that game designers look for the “play space” in the topic, the types of things experts in the field mull over in their spare time; whilst game design veterans, Brathwaite and Schreiber (2009) suggest that games can be made quite easily from systems, so they suggest that game designers look for the systems in the learning to adapt to game format. In addition to this, we also believe that games designers should also consider emotional design objectives (e.g., what do you want players to feel empathy with/disgust for) and explore ways the elements of a game could foster these emotions in players.

Two of the four mini-games in *Orbit* have some components based on a quiz mechanic. In part, this was because many of our learning objectives are fact-based and we were using a rules-based approach to understanding sexual abuse. However, the adventure game component of the game (the main storyline) does go beyond rote learning and all of the mini-games do have game-play elements that go beyond rote learning. If a game needs to resort to using quiz-like mechanics, players should be given opportunities to correct their mistakes in a meaningful way that goes beyond brute-force methods. Where we have adopted a quiz mechanic in *Orbit*, we do not simply provide direct and immediate feedback, but instead the correct solutions are uncovered through exploration and game play.

Learning game designers have more opportunity for creating games that go beyond rote learning when the game is viewed as being integrated into a wider learning program, not just as a standalone resource (Klopfer et al., 2009).

### Consider how the player can make the world their own and let them share it with others

In order to encourage players to personalize game learnings, look for opportunities to help the player make the game their own. In *Orbit* we did this through encouraging players to build the player character avatar in their own likeness and trusted adult avatars in the likeness of the adults in their life. *Orbit* also allowed the player to customize a subset of game-rooms and gave class-mates the opportunity to see how other players customized their game rooms by virtually “visiting” their spaceship. These types of sharing opportunities are motivating for some players. When facilitating sharing opportunities, player safety must be considered, especially for games aimed at children. To this end, *Orbit* only allowed students from within class groupings to visit one another's spaceships. We also limit communication between classmates and trusted adults through sharing of predefined positive messages.

Other game customization tools used by entertainment games include level editors (e.g., Ubisoft Entertainment, 2011), game photo album utilities (e.g., Heliö, 2005) and videoing game play (Ubisoft Entertainment, 2011). These tend to be used as motivational tools for players. Sometimes the game developer also provides the capacity for the player to share the content they created online.

### Conduct rigorous evaluation

Serious Games should be evaluated both against their learning objectives and for the quality of the game-play. Learning objectives should be measurable in some way. We used a variation on Bloom's taxonomy of educational objectives: cognitive domain (Bloom et al., 1956; James, 2008) to ensure that our learning objectives were measurable. Interestingly, we found that many of our learning objectives tended to be knowledge and comprehension sections of Bloom's taxonomy which are the least complex areas of cognitive domain; and this is possibly why we found quiz-based game mechanics most suited to some of the game learnings.

Unpublished research of the authors indicates that some teachers use games in their curriculum to emotionally engage students with a topic thus allowing the teacher to build on this engaging platform using other classroom activities. Therefore, learning game designers should also consider affective and user experience objectives when designing learning games.

Finally, we recommend, where possible, to use pre-existing, standardized measures when evaluating learning games and to consider employing comprehensive evaluation methodologies which may include a control group and pre- and post-tests. In addition to a comprehensive evaluation design, we also decided to include a separate play-test group to investigate the quality of the game-play thoroughly.

### Conflict of interest statement

The authors declare that the research was conducted in the absence of any commercial or financial relationships that could be construed as a potential conflict of interest.

## References

[B1] Access Economics. (2008). The Cost of Child Abuse in Australia: Report by Access Economics Pty Limited, Australian Childhood Foundation and Child Abuse Prevention Research Australia at Monash University. Available online at: http://www.childhood.org.au/research/

[B2] Alamri A. Hassan M. M. Hossain M. A. Al-Qurishi M. Aldukhayyil Y. Hossain M. S. (2014). Evaluating the impact of a cloud-based serious game on obese people. Comput. Hum. Behav. 30, 468–475 10.1016/j.chb.2013.06.021

[B3] Asdigian N. L. Finkelhor D. (1995). What works for children in resisting assaults? J. Interpers. Violence 10, 402–418 10.1177/088626095010004002

[B4] Beale I. L. Kato P. M. Marin-Bowling V. M. Guthrie M. S. Cole S. W. (2007). Improvement in cancer-related knowledge following use of a psychoeducational video game for adolescents and young adults with cancer. J. Adolesc. Health 41, 263–227 10.1016/j.jadohealth.2007.04.006 17707296

[B5] Beale I. L. Marin-Bowling V. M. Guthrie M. S. Kato P. M. (2006). Young cancer patients' perceptions of a video game used to promote self care. Int. Electron. J. Health Educ. 9, 202–212

[B6] Berkeley L. (2006). Situating machinima in the new mediascape. Austr. J. Emerging Technol. Soc. 4, 65–80

[B7] Berrick J. D. Barth R. P. (1992). Child sexual abuse prevention: research review and recommendations. Soc. Work Res. Abstr. 28, 6–15 10.1093/swra/28.4.6

[B8] Bloom B. S. Engelhart M. Furst E. J. Hill W. H. Krathwohl D. R. (1956). Taxonomy of Educational Objectives: Handbook I: Cognitive Domain. New York, NY: David McKay

[B9] Brand J. E. Borchard J. Holmes K. (2008). Interactive Australia 2009. Gold Coast, QLD: Bond University

[B10] Brathwaite B. Schreiber I. (2009). Challenges for Game Designers: Non-Digital Exercises for Video Game Designers. Boston, USA: Cengage Learning

[B11] Briggs F. Hawkins R. M. (1994). Follow-up data on the effectiveness of New Zealand's national school based child protection program. Child Abuse Negl. 18, 635–643 10.1016/0145-2134(94)90013-2 7953903

[B12] Briggs F. McVeity M. (2003). Teaching Children to Protect Themselves. Crows Nest, NSW: Allen and Unwin

[B13] Brown S. L. (2009). Play: How It Shapes the Brain, Opens the Imagination, and Invigorates the Soul. New York, NY: Avery/Penguin Group

[B14] Budin L. E. Johnson C. F. (1989). Sex abuse prevention programs: offenders' attitudes about their efficacy. Child Abuse Negl. 13, 77–87 10.1016/0145-2134(89)90031-8 2706563

[B15] Crookall D. (2010). Serious games, debriefing, and simulation/gaming as a discipline. Simul. Gaming 41, 898–920 10.1177/1046878110390784

[B16] Currier L. L. Wurtele S. K. (1996). A pilot study of previously abused and non-sexually abused children's responses to a personal safety program. J. Child Sex. Abus. 5, 71–87 10.1300/J070v05n01_04

[B17] de Freitas S. Oliver M. (2006). How can exploratory learning with games and simulations within the curriculum be most effectively evaluated? Comput. Educ. 46, 249–264 10.1016/j.compedu.2005.11.007

[B18] De Freitas S. Rebolledo-Mendez G. Liarokapis F. Magoulas G. Poulovassilis A. (2010). Learning as immersive experiences: using the four-dimensional framework for designing and evaluating immersive learning experiences in a virtual world. Br. J. Educ. Technol. 41, 69–85 10.1111/j.1467-8535.2009.01024.x

[B19] Dietz T. L. (1998). An examination of violence and gender role portrayals in video games: implications for gender socialization and aggressive behavior. Sex Roles 38, 425–442 10.1023/A:1018709905920

[B20] Dondlinger M. J. (2007). Educational video game design: a review of the literature. J. Appl. Educ. Technol. 4, 21–31

[B21] Egenfeldt-Nielsen S. (2005). Beyond Edutainment: Exploring the Educational Potential of Computer Games. Copenhagen: IT-University of Copenhagen

[B22] Ekanayake H. Backlund P. Ziemke T. Ramberg R. Hewagamage K. (2010). Game interaction state graphs for evaluation of user engagement in explorative and experience-based training games, in Paper Presented at 2010 International Conference on the Advances in ICT for Emerging Regions (ICTer). (Colomba).

[B23] Eklund K. McGonigal J. Cook D. Lamb M. Senderhauf M. Wells K. (2007). World Without Oil. Free Online Multiplayer Educational Game, Independent Lens, s. unter Available online at: http://www.worldwithoutoil.org/[8.10. 2010]

[B24] Finkelhor D. (1999). Child sexual abuse. challenges facing child protection and mental health professionals, in Childhood and Trauma: Separation, Abuse, War, eds Ullmann E. Hilweg W. (Aldershot: Ashgate Publishing), 101–116

[B25] Finkelhor D. Asdigian N. Dziuba-Leatherman J. (1995). Victimization prevention programs for children: a follow-up. Am. J. Public Health 85, 1684–1689 10.2105/AJPH.85.12.1684 7503345 PMC1615746

[B26] Finkelhor D. Dziuba-Leatherman J. (1995). Victimization prevention programs: a national survey of children's exposure and reactions. Child Abuse Negl. 19, 129–139 10.1016/0145-2134(94)00111-7 7780776

[B27] Finkelhor D. Strapko N. (1992). Sexual abuse prevention education: a review of evaluation studies, in Prevention of Child Maltreatment: Developmental and Ecological Perspectives, eds Willis D. J. Holden E. W. Rosenburg S. (New York, NY: John Wiley & Sons), 150–167

[B28] Froschauer J. Siedel I. Gartner M. Berger H. Merkl D. (2010). Design and evaluation of a Serious Game for immersive cultural training, in Paper Presented at the 2010 16th International Conference on Virtual Systems and Multimedia (VSMM). (Seoul).

[B29] Games for Change. (2005). Accessed March 22, 2013. Available online at: http://www.gamesforchange.org/

[B30] Games for Health. (2013). Accessed March 22, 2013. Available online at: http://gamesforhealth.org/

[B31] Gee J. P. (2003). What Video Games Have to Teach us about Literacy and Learning. New York, NY: Palgrave Macmillan

[B32] Gee J. P. (2008). Learning and Games, in The Ecology of Games: Connecting Youth, Games, and Learning, ed Salen K. (Cambridge, MA: The MIT Press), 21–40

[B33] Gibson D. Aldrich C. Prensky M. (2007). Games and Simulations in Online Learning: Research and Development Frameworks. Hershey, PA: Information Science Publishing

[B34] Hagen C. (2013). SimCity Breaks Ground in the Classroom. Available online at: http://www.ea.com/news/simcity-breaks-ground-in-the-classroom

[B35] Hazzard A. Webb C. Kleemeier C. Angert L. Pohl J. (1991). Child sexual abuse prevention: evaluation and one-year follow-up. Child Abuse Negl. 15, 123–138 10.1016/0145-2134(91)90097-W 2029665

[B36] Hedden C. (1998). A Guided Exploration Model of Problem-Solving Discovery Learning. University of Washington

[B37] Heliö S. (2005). Simulating the storytelling qualities of life: telling stories with the sims, Paper presented at the DIGRA 2005 International Conference, Simon Fraser University (Vancouver, BC).

[B38] James J. (2008). Writing Good Learning Objectives. Accessed April 29, 2013. Available online at: http://www.scribd.com/doc/17374092/Writing-Good-Learning-Objectives-Handout

[B39] Johnson C. F. (2004). Child sexual abuse. Lancet 364, 462–470 10.1016/S0140-6736(04)16771-8 15288746

[B40] Kato P. M. Cole S. W. Bradlyn A. S. Pollock B. H. (2008). A video game improves behavioral outcomes in adolescents and young adults with cancer: a randomized trial. Pediatrics 122, e305–e317 10.1542/peds.2007-3134 18676516

[B41] Kelly H. Howell K. Glinert E. Holding L. Swain C. Burrowbridge A. (2007). How to build serious games. Commun. ACM 50, 44–49 10.1145/1272516.127253817880680

[B42] Kirriemuir J. McFarlane A. (2004). Report 8: Literature Review in Games and Learning. Bristol: Futurelab

[B43] Klopfer E. Osterweil S. Salen K. (2009). Moving Learning Games Forward: Obstacles, Opportunities and Openness (White Paper). Cambridge, MA: The Education Arcade, Massachuesetts Institute of Technology

[B44] Knight J. F. Carley S. Tregunna B. Jarvis S. Smithies R. de Freitas S. (2010). Serious gaming technology in major incident triage training: a pragmatic controlled trial. Resuscitation 81, 1175–1179 10.1016/j.resuscitation.2010.03.042 20732609

[B45] Kognito Interactive. (2009). At-Risk: Identify and Refer Students in Mental Distress: Results from a National Study in 42 Leading Universities in the U.S. New York, NY: Kognito Interactive

[B46] Kriz W. C. (2010). A systemic-constructivist approach to the facilitation and debriefing of simulations and games. Simul. Gaming 41, 663–680 10.1177/1046878108319867

[B47] Lamont A. (2011). Who Abuses Children. Available online at: http://www.aifs.gov.au/nch/pubs/sheets/rs7/rs7.html

[B48] Malone T. W. (1980). What makes things fun to learn? heuristics for designing instructional computer games, in Paper Presented at the Proceedings of the 3rd ACM SIGSMALL Symposium and the First SIGPC Symposium on Small Systems. (Palo Alto, CA). 10.1145/800088.802839

[B49] Mayer I. Bekebrede G. Harteveld C. Warmelink H. Zhou Q. van Ruijven T. (2013). The research and evaluation of serious games: toward a comprehensive methodology. Br. J. Educ. Technol. 10.1111/bjet.12067

[B50] Melton G. B. Flood M. F. (1994). Research policy and child maltreatment: developing the scientific foundation for effective protection of children. Child Abuse Negl. 18, 1–28 10.1016/0145-2134(94)90088-4 8082045

[B51] Moreno R. Mayer R. (2007). Interactive multimodal learning environments. Educ. Psychol. Rev. 19, 309–326 10.1007/s10648-007-9047-2

[B52] Moreno R. Mayer R. E. (1999). Cognitive principles of multimedia learning: the role of modality and contiguity. J. Educ. Psychol. 91, 358–368 10.1037/0022-0663.91.2.358

[B53] Mullen P. E. Fleming J. (1998). Long-term effects of child sexual abuse. Issues Child Abuse Prev. 9 Available online at: http://www.aifs.gov.au/nch/pubs/issues/issues9/issues9.html

[B54] Muratet M. Torguet P. Viallet F. Jessel J. P. (2011). Experimental feedback on prog and play: a serious game for programming practice. Comput. Graphics Forum 30, 61–73 10.1111/j.1467-8659.2010.01829.x

[B55] Nacke L. Drachen A. Göbel S. (2010). Methods for evaluating gameplay experience in a serious gaming context. Int. J. Comput. Sci. Sport 9, 1–12

[B56] Nudell H. Brunner C. Panik S. (2007). Playing 4 Keeps Evaluation Report. (Waltham, MA).

[B57] Paavilainen J. Kultima A. Kuittinen J. Mayra F. Saarenpaa H. Niemela J. (2009). GameSpace: Methods for Design and Evaluation for Casual Mobile Multiplayer Games. Tampere: University of Tampere

[B58] Poremba C. (2003). Remaking Each other's Dreams: Player Authors in Digital Games. Vancouver, BC: New Forms Festival ‘03, Canada

[B59] Putnam F. W. (2003). Ten-year research update review: child sexual abuse. J. Am. Acad. Child Adolesc. Psychiatry 42, 269–278 10.1097/00004583-200303000-00006 12595779

[B60] Queensland Government (2011). Child Sexual Abuse. Accessed May 23, 2011. Available online at: http://www.communities.qld.gov.au/childsafety/protecting-children/what-is-child-abuse/child-sexual-abuse

[B61] Queensland Government (2012). Protective Behaviours for Children. Accessed March 30. 2013, Available online at: http://www.police.qld.gov.au/programs/cscp/personalSafety/children/Protective+Behaviours+For+Children.htm

[B62] Rispens J. Aleman A. Goudena P. P. (1997). Prevention of child sexual abuse victimization: a meta-analysis of school programs. Child Abuse Negl. 21, 975–987 10.1016/S0145-2134(97)00058-6 9330798

[B63] Salen K. Zimmerman E. (2004). Rules of Play: Game Design Fundamentals. Cambridge, MA: The MIT Press

[B64] Sanderson J. (2004). Child-Focused Sexual Abuse Prevention Programs: How Effective are They in Preventing Child Abuse? Brisbane, QLD: Crime and Misconduct Commission

[B65] Sawyer B. Smith P. (2008). Serious Game Taxonomy. Portland, ME: Serious Games Initiative

[B66] Schell J. (2008). The Art of Game Design: A Book of Lenses, 1 Edn. Burlington, MA: Morgan Kaufmann Publishers

[B67] Scholes L. Jones C. Stieler-Hunt C. Rolfe B. Pozzebon K. (2012). The teachers' role in child sexual abuse prevention programs: implications for teacher education. Austr. J. Teacher Educ. 37, 6 10.14221/ajte.2012v37n11.5

[B68] Shute V. J. Masduki I. Donmez O. Kim Y. J. Dennen V. P. Jeong A. C. (2010). Modeling, assessing, and supporting key competencies within game environments, in Computer-Based Diagnostics and Systematic Analysis of Knowledge, eds Ifenthaler D. Pirnay-Dummer P. Seel N. M. (New York, NY: Springer-Verlag), 281–309 10.1007/978-1-4419-5662-0_15

[B69] Smallbone S. W. Wortley R. K. (2001). Child Sexual Abuse: Offender Characteristics and Modus Operandi, Vol. 193 Canberra, ACT: Australian Institute of Criminology

[B70] Solomon J. McGonigal J. Yost J. Broome R. (2012). SuperBetter. Accessed April 29, 2013. Available online at: https://www.superbetter.com/about

[B71] Somer E. Szwarcberg S. (2001). Variables in delayed disclosure of childhood sexual abuse. Am. J. Orthopsychiatry 71, 332–341 10.1037/0002-9432.71.3.332 11495335

[B72] Swain C. (2007). Designing games to effect social change, in Paper presented at the DiGRA 2007 Conference (Tokyo: Situated Play).

[B73] Thomas G. M. (2004). Building the buzz in the hive mind. J. Consum. Behav. 4, 64–72 10.1002/cb.158

[B74] Tucci J. Mitchell J. Goddard C. (2006). Out of Sight-Out of Mind: Community Attitudes about Child Abuse and Child Protection in Australia. Ringwood Victoria: Australian Childhood Foundation

[B75] Tutty L. M. (1995). The revised children's knowledge of abuse questionnaire: development of a measure of children's understanding of sexual abuse prevention concepts. Soc. Work Res. 19, 112–120

[B76] Ubisoft Entertainment. (2011). Track Mania Forever. Accessed April 29, 2013. Available online at: http://trackmaniaforever.com/

[B82] Wrzesien M. Alcañiz Raya M. (2010). Learning in serious virtual worlds: evaluation of learning effectiveness and appeal to students in the E-Junior project. Comput. Educ. 55, 178–187 10.1016/j.compedu.2010.01.003

[B78] Wurtele S. K. (2002). School-based child sexual abuse prevention, in Preventing Violence in Relationships: Interventions across the Life Span, ed Schewe P. A. (Washington, DC: American Psychological Association), 9–25 10.1037/10455-001

[B79] Wurtele S. K. (2009). Preventing sexual abuse of children in the twenty-first century: preparing for challenges and opportunities. J. Child Sex. Abus. 18, 1–18 10.1080/10538710802584650 19197612

[B80] Wurtele S. K. Hughes J. Owens J. S. (1998). An examination of the reliability of the “What if” situations test: a brief report. J. Child Sex. Abus. 7, 41–52 10.1300/J070v07n01_03

[B81] Zyda M. (2005). From visual simulation to virtual reality to games. IEEE Comput. 38, 25–32 10.1109/MC.2005.297

